# Airborne vocal communication in adult neotropical otters (*Lontra longicaudis*)

**DOI:** 10.1371/journal.pone.0251974

**Published:** 2021-05-26

**Authors:** Sabrina Bettoni, Angela Stoeger, Camilo Rodriguez, W. Tecumseh Fitch

**Affiliations:** Department of Behavioral and Cognitive Biology, University of Vienna, Vienna, Austria; University of Sussex, UNITED KINGDOM

## Abstract

Most aquatic mammals have complex social and communication systems. Interestingly, little is known about otters’ vocal communication compared to other aquatic mammals. Here, for the first time, we acoustically describe vocalizations of the neotropical otter (*Lontra longicaudis*), a solitary and endangered New World otter species. We recorded vocalizations and behavioral contexts from six captive neotropical otters at Projeto Lontra, Santa Catarina Island, Brazil. Analysis of acoustic parameters were used to classify the vocalizations according to structure and context. We describe six call types with highly tonal as well as chaotic vocalizations with fundamental frequencies ranging from 90 to 2500 Hz. Additionally, we identified sex differences in the usage of calls. Results suggest that the neotropical river otter has a rich vocal repertoire, similar in complexity to other solitary otter species, but less complex than that of the social giant otter. Despite differences in sociality, phylogeny and ecology, *L*. *longicaudis* seems to possess vocalizations homologous to those found in other otters (e.g. hah and chirp), suggesting phylogenetic inertia in otter communicative repertoire. Otters thus offer an interesting but neglected group to explore the evolution of communication systems.

## Introduction

Vertebrate life originated in the water, and multiple mammal groups have independently returned to an aquatic or semi-aquatic lifestyle including cetaceans, pinnipeds, otters and some rodents. Aquatic mammals often have complex social lives and communication systems. Otters are a clade of carnivores highly adapted to aquatic life (second only to the pinnipeds: seals and walruses), whose communication system remains relatively unstudied. Otters range in social system from relatively solitary (e.g. European river otters, *Lutra lutra*) to highly social and gregarious (sea otters, *Enhydra lutris* or giant otters, *Pteronura brasiliensis*). Otters can be highly vocal, and exhibit considerable diversity in their vocalizations. Vocal communication is present in situations of conflict avoidance, survival, mating, and parental care, among others [1–4]. Some otter species have up to 22 call types in their vocal repertoire and show individual and group signatures in their calls and/or possess complex choruses [2–5], while other species live mostly solitary lives and have a much less complex vocal repertoire, down to four call types [6–8]. Understanding aspects of otter vocal communication, particularly from a comparative perspective, across otter species and in relation to their aquatic and terrestrial relatives, can thus contribute significantly to increasing knowledge of otter social behavior, evolution, and ecology. As a group, otters thus offer an interesting and neglected group in which to explore the evolution of social behavior and communication systems, and provide a comparison to the better-studied cetaceans and seals.

The otters comprise the carnivore subfamily Lutrinae, with 13 otter species, providing a promising group to study the diversity of vocal communication. These semi-aquatic carnivores are found in every continent, except Antarctica and Australasia [9]. The subfamily Lutrinae is monophyletic, and resolved into three primary clades: one containing the Old World river otters and the sea otter (*Aonyx*, *Lutrogale*, *Lutra*, *Enhydra* and *Hydrictis*), a second containing New World river otters (*Lontra*) and a third containing the monotypic giant otter (*Pteronura*) [10].

Among otter species, the giant otter is the most social and the most vocal of the studied species [2,11]. Leuchtenberger et al. [2] described the airborne vocal repertoire of nine giant otters in the Pantanal area of Brazil, comprising 15 call types emitted in different behavioral contexts. Nonlinear phenomena (NLP) were observed in screams and high screams in adult giant otters. NLP include biphonation, subharmonics, and deterministic chaos and are characterized by aperiodic and chaotic vocal fold oscillations, and are common in most of mammalian species’ vocal repertoires and may play a key role in mammal vocal communication systems [12,13]. Calls containing NLP may contribute to signaling motivation and status [14], and/or function in sexual selection [15]. Further, for the giant otter several underwater calls have been described [16], however, a more recent study only found one distinct underwater call, that may have a cohesion function [17]. Underwater acoustic communication has not been well explored in other otter species.

Studies examining the vocal repertoire of North American river otters (*Lontra canadensis*) investigating 10 [7] and 12 [6] individuals of captive river otter suggested that this otter species has a vocal repertoire comprising four distinct vocal types and seven sub-call types. McShane et al. [18] studied the vocal behavior of sea otters (*Enhydra lutris*) based on recordings from wild and captive adults and young. Their vocal repertoire of 10 call types was described as being more complex than those of North American river otters with four call types, but less than that of giant otters, with 15 or 22 call types, and similar in complexity, in terms of call types, to most pinnipeds. The Eurasian otter is the least social of all the otters [19]. A study on Eurasian otter vocal communication identified seven call types and specific behavioral contexts [7]. Lemmason and colleagues [1] described aspects of vocal communication of Asian small-clawed otter (*Aonyx cinerea*), finding that their vocal repertoire consists of four context-dependent vocalization units divided into seven vocalization types. Finally, the neotropical river otter has been reported to utter whistles and an inquisitive “hahh” call [20,21], but these seem to be the only published report concerning neotropical otter vocal communication, and include no thorough description or acoustic information about these calls. Studies of vocal communication in the remaining otter species are limited or non-existent.

So far there are no detailed studies of vocal communication in the neotropical otter; as reviewed above, existing studies mostly focus on highly social species (e.g. giant otter and Asian short-clawed otter). By studying the vocal communication of the solitary neotropical otter we increase the variety of known social systems in otter species. The current paper represents a detailed study of the vocal behavior in neotropical river otters (*Lontra longicaudis* Olfers, 1818). We observed and recorded individuals in a captive environment, allowing close range observation, and enabling us to gain important new information about a species that is difficult to observe in its natural habitat. The elusive nature of this species might be one reason for the lack of published knowledge of their vocal behavior.

Neotropical otters are listed as endangered in the Appendix I of the Convention on International Trade of Endangered Species of Wild Fauna and Flora (CITES), and classified as “near threatened” in Brazil according to the Red Book of the Brazilian Endangered Fauna [22]. Its genus, *Lontra*, comprises four species of New World otters [23]. The neotropical river otter is one of five species of the sub-family Lutrinae living in the neotropics. The species is widely distributed, occurring from Mexico, Central America south to Uruguay in South America [24,25]. This very large geographical range covers several climates and different habitats including rivers, lakes marshes, sea coasts and coastal islands, as well as mangroves and wetlands [26,27]. *L*. *longicaudis* can live solitarily or in pairs [20], and the solitary and territorial females are tolerant of males’ presence only during breeding. Neotropical otters have no specific breeding season and they can produce one to five cubs per litter [21,26,28]. Previous studies of the neotropical otter have been largely focused on diet, ecology and distribution [29,30].

The current study aims (1) to quantitatively describe the airborne vocal repertoire of captive neotropical river otters and (2) to associate call types with their behavioral context. In addition, (3) we compare our results with the known literature to investigate possible effects of social system and/or phylogeny on vocal behavior. We provide here a thorough description of the neotropical river otter vocal communication system. Information on vocal communication in this otter species can benefit conservation and management efforts, and may potentially be used for non-invasive acoustic monitoring and censusing.

## Methods

### Study site and animals

We recorded vocalizations and observed behavior in six captive adult neotropical otters (three males, three females), housed in pairs in three different enclosures at the Instituto Ekko Brasil, Projeto Lontra Research Station in Santa Catarina Island, Brazil (27°44’08.4"S 48°30’57.2"W). Data was collected in two different data collection sessions, first from September 2013 to December 2014 (behavioral observations), and the second one in January 2017 (audio recordings). During the first data collection period 2013/2014 the six adults, three males and three females, were housed in male/female pairs in three different enclosure located in a row (see Table in S1 Table). The animals had auditory contact with all the other otters but only partial visual and physical contact depending on their enclosure. Their housing conditions and group composition changed between the two data collection periods because one adult female died. The other otters were then placed into new enclosures, rearranged into different pairs, and one male was housed alone. The individuals in these new enclosures had auditory contact with all the other otters but restricted visual contact and no physical contact. The study site is open to visitors from 8am to 10am and from 4pm to 6pm (overlapping with our recording times). The animals were fed twice daily between 9am and 10am, and between 4pm to 5pm (occasionally overlapping with our recording times).

### Data collection

#### Behavioral data collection

The first data collection period from September 2013 to December 2014 focused on understanding the behavioral context of the otters’ vocalizations. It had an exploratory initial phase in which we recorded data from the six otters, one enclosure at a time, using observational group sampling (sampling rule: group sampling; recording rule: continuous recording, session length of one hour) for 14 hours in total, over September 2013 to November 2013 to establish an initial list of vocalizations uttered by those individuals, and an ethogram with the behavioral repertoire associated with their vocal communication.

We used a chronometer and a spreadsheet on paper which we filled out manually during direct observations. For every hour session we recorded behavioral observations for every call occurrence from the otters housed in one enclosure, and noted the call type, its associated behavior(s), identity of signaler and identity of potential receiver (individual from either the same or other enclosures and people), as determined by the emitter’s head or gaze direction during calling. We additionally ran an MP3 recorder for control purpose, so in case of any doubt during the data collection we could go back to the audio recordings (these were not acoustically analyzed). Our initial call classification, before running any statistical tests was based on visual inspection of sonograms and aural distinction of the vocalizations. We named the distinct neotropical otter’s calls according to their sound structure as perceived by the observer and with reference to the classification of other otter species’ vocal repertoire by previous authors. We identified 46 associated behaviors which were further grouped in 14 behavioral categories. This might not constitute a complete ethogram of the neotropical otter vocal communication, since there may be more behaviors not observed in this study. We then recorded behavioral observations of the six otters for 69 hours over 10 months, from March until December 2014, using an observational group sampling method, the same used for the initial exploratory phase. We collected in average 2.43 (SD + 1.19) hours of data per day. Among the total number of 69 sessions, we collected 23 hours of data from enclosure I, 29 hours from enclosure II and 11 hours from enclosure III (more information on housing see Table in S1 Table). We used the same approach as for the exploratory phase but now used the 46 associated behaviors, established during the exploratory phase, to consistently fill in the spreadsheet information for call-associated behavior.

Each observation session lasted one hour. We recorded one enclosure at a time, with two individuals being recorded at the same time. The observations were conducted between 7am to 10am and 3pm to 6pm (adjusted to remain invariant through daylight saving time changes), the diurnal period when the animals are most active. The order of observations was randomized per enclosure however, other factors (e.g. animals’ sleep pattern and health, disturbance by visitors) could affect the order. Data was not collected during periods or days of heavy rain. We could not record the animals during night or crepuscular hours. A single observer (SB) collected all data throughout the study. The distance from the observer to the animal was between two and 15 meters, and the observer had free access to the enclosures’ surroundings so that the otters could be easily observed.

#### Audio recordings

We recorded 26 hours of airborne vocalizations during the second data collection period in January 2017 with a Sennheiser ME-67 directional microphone mounted on a Rycote Modular Windshield WS 7 Kit for Shotgun Microphones (frequency response 40–20000 Hz, ± 2.5 dB; Sennheiser Electronic GmbH, Wedemark, Germany), connected to a Zoom H4N digital recorder (48 kHz sampling frequency and 16-bit quantization; Zoom Corporation, Tokyo, Japan). The recordings were stored as uncompressed WAV files. The otter producing each vocalization was individually identified based on call direction. We used a chronometer and a spreadsheet on paper, which we filled out manually. We recorded one enclosure at a time using observational group sampling. The data collection depended on the possibility of access to the observation and data collection area of each enclosure, so it could not be randomized between enclosures. The recorded 26 hours were balanced between enclosure II and III, with 12.5 hours each, and unbalanced compared to enclosure I with one hour of data collection. We recorded the data between 7am to 10am and 3pm to 6pm. Data was not collected during periods or days of heavy rain. A single observer (SB) recorded all data throughout the study.

#### Animal welfare

This non-experimental research meets all applicable European Union and Brazilian laws and was conducted in accordance with the Guidelines for the Treatment of Animals in Behavioural Research and Teaching [31]. All participating institutions mentioned in this manuscript approved data collection for the study. The nature of the study was purely observational: No invasive methodologies were applied at any point of the study. The research did not affect the housing, daily routine, behavior, diet or management of the animals. Therefore, no ethics committee approval was required.

### Data analysis

#### Extraction of acoustic parameters

We selected the highest quality calls from the sound files, excluding calls that overlapped with other calls, and/or which contained significant background noise. We manually annotated these calls, notating call type and individual identity using the ‘Annotate: To TextGrid’ function within Praat [32]. From this we obtained 422 annotated calls for subsequent extraction of source- related parameters and acoustic analysis.

Source-related parameters are those relating to vibrations in the laryngeal source, particularly fundamental frequency (f_0_) (which corresponds to the rate of vocal fold vibration) and nonlinearities (which correspond to the irregularity of vocal fold vibrations, and potential inclusion of other vibratory tissue within the larynx: see Fitch et al. [12]). We extracted information and sound parameters from the calls mainly using a custom-built Praat script written by the authors or occasionally manually in Praat. The script opened the annotated audio files, specified a set of input parameters for each call type (see S2 Table), and semi-automatically extracted the following acoustic parameters: mean fundamental frequency (f_0_), minimum f_0_, maximum f_0_, dominant frequency (highest amplitude frequency in the spectrum), standard deviation of f_0_ (standard deviation of f_0_ within call duration), f_0_ at the start of the call (initial f_0_), f_0_ at the time of mid-point of the call (mid-point f_0_), f_0_ at the end-point of the call (end f_0_), f_0_ slope (rate of change of f_0,_ in units of Hz per second) for the first and second half of the call, f_0_ range and call duration. We used the custom-built Praat script to extract these acoustic parameters from the call types chirp, chuckle, growl and squeak.

The irregular calls (scream and hah) contained either no clear fundamental frequency or only short segments of clear f_0_ during the call. For these calls we manually extracted dominant frequency, call duration, and we quantified the occurrence of the nonlinear phenomena (NLP), identified visually from the spectrogram following [12], by measuring the duration of chaos, subharmonics and/or biphonation in proportion to the duration of the entire call.

#### Statistical analysis

To determine the association between the call types, behavioral context and sex, we performed a Multiple Correspondence Analysis (MCA) using the “MCA” function in the “FactoMineR” package [33] in the R software package [34]. MCA is a multivariate analysis that aims to identify associations among several (more than two) categorical variables. MCA clusters data points (observations) based on a similarity matrix in a multidimensional Euclidean space. That is, the more similar are the data points within categories, the closer they are in the Euclidean space [35]. Compared to the more familiar PCA, which applies only to normally distributed quantitative variables, MCA makes fewer assumptions about the data, and supports the use of nominal, categorical data along with quantitative data. MCA does not require meeting any distributional or other underlying assumptions [36–38]. For this analysis we used the variables from the behavioral observations, named call type, its associated behavior(s), sex of signaler and identity of potential receiver. Subsequently, in order to determine how behaviors, call types and sexes clustered together, we performed a Hierarchical clustering on principle components (HCPC) with cumulative proportion of explained variance greater than 80%, using the “HCPC” function in the “FactoMineR” package [33] in the R software [39].

To evaluate differences in the acoustic parameters between call types, we first performed a Varimax normalized Principal Component Analysis (PCA) to reduce redundancy between the acoustic parameters, for the tonal and the irregular NLP-containing calls separately, using the “principal” function in the “psych” package [40] in R. The resultant principal components with eigen values greater than 1 were then used as explanatory variables in a Kruskal-Wallis test to evaluate acoustic differences between call types. Subsequently, we performed Pairwise comparisons between call types using Wilcoxon rank sum tests with Bonferroni correction. We calculated the emission rate, number of calls per each session (one hour) and its mean and standard deviation, for each call type and sex.

## Results

**Fig 1:**
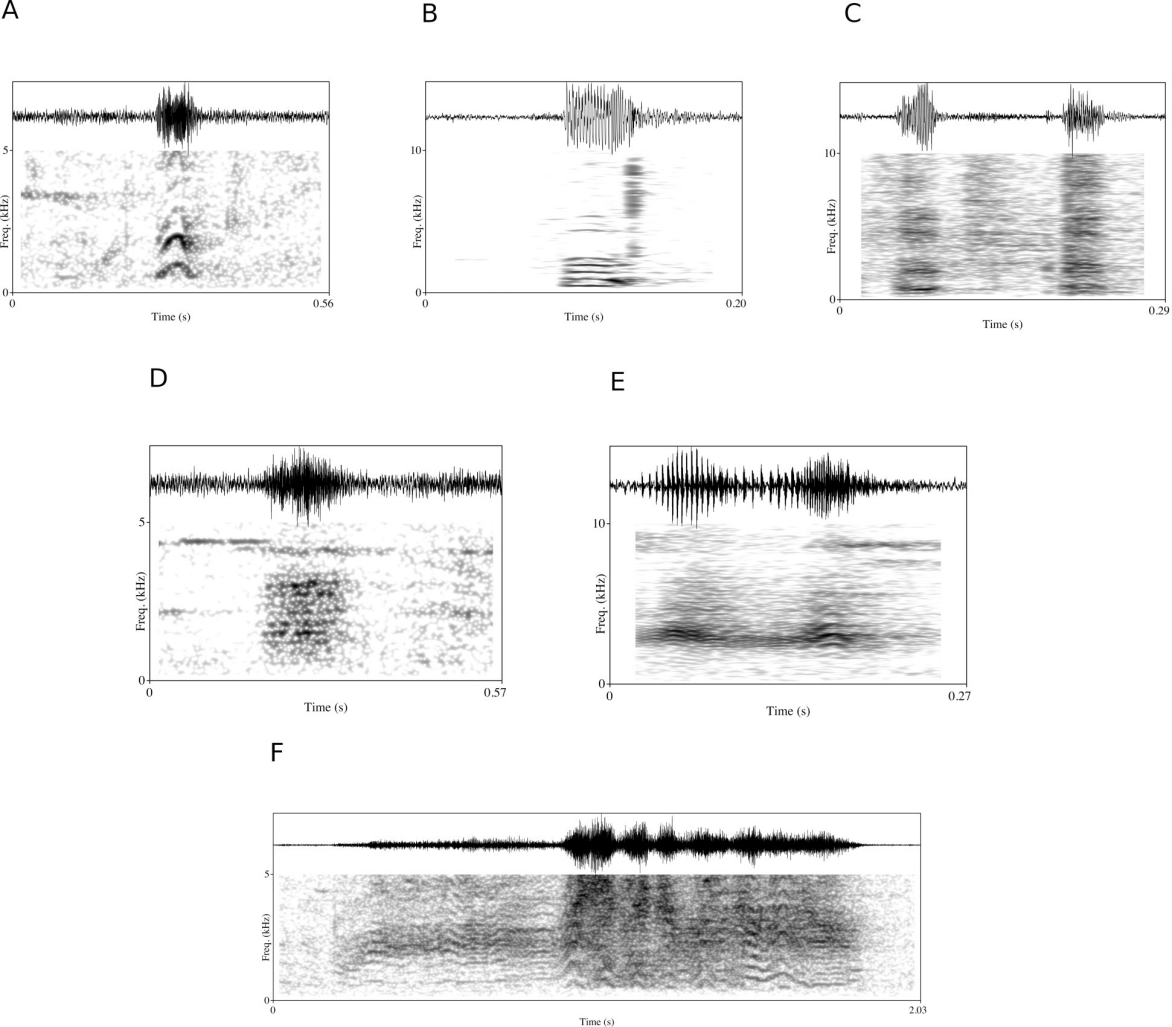
**Spectrograms and oscillograms (top) of vocalizations emitted by neotropical river otters.** (A) Chirp, (B) squeak, (C) chuckle, (D) hah, (E) growl, (F) scream.

We used 422 high-quality recorded calls, along with 990 call occurrences including behavioral context to establish the vocal repertoire of the neotropical river otter. The vocal repertoire was classified into six call types: chirps, squeaks, chuckles, growls, hahs and screams (Fig 1). We identified 14 behavioral categories associated with this vocal repertoire (Table 1). Our initial auditory classification of the vocal repertoire comprised seven call types, however, based on later acoustic and statistical analysis, we re-classified two initial aurally-determined scream types (short and long screams) into one graded type (screams), yielding six clearly distinguishable call types.

**Table 1:** Behavioral categories and descriptions associated with the vocal repertoire of the neotropical river otter.

Grouped category	Category of behavior	Behavior
**Social Agonistic**	**Physical attack.** Physical attack on another otter.	Physical attack on another otter of the same enclosure by biting, scratching or violent wrestling.
		Physical attack on another otter of adjacent enclosure by biting, scratching or violent wrestling.
	**Defending from attack.** Defending self while another otter displayed direct agonistic behavior.	Defending itself by either crouching with its head down or turned towards the attacker with its head up and mouth slightly opened showing teeth, while another otter displayed direct agonistic behavior
	**Protecting food.** Either threatening another otter or moving away to protect its food.	Eating and increasing distance from another otter (that might be approaching).
		Running away from another otter with fish in its mouth.
		Turn body and move briskly towards the other otter while either vocalizing or showing threatening body posture to defend its food, during an attempt to steal food by another otter.
		Holding its food (feeding time) warning with vocalization and gazing at the other otter (or human).
	**Reject/complain.** Display discontent with proximity of another otter (or human).	Showing discontent of the proximity of an individual from the adjacent enclosure by approaching the fence with an aggressive body posture and directed eye gaze.
		Reacting to another otter touching it (or approached too close), and avoiding interaction by turning head, neck and gaze while showing aggressive body posture and/or vocalizing and slightly showing teeth.
		Anxiously running to get food during feeding time and bumping into the other otter.
		Interacting agonistically with human observer, moving towards them with aggressive body posture.
		Reacting to human approach or proximity by either distancing away or orienting its body towards the person with a staring gaze.
		Reacting to another individual during social play in the water by turning its head towards the other otter, opening mouth, showing teeth and/or softly biting the other otter.
		Reacting (mild complaint) while being groomed by quickly turning its head towards the other otter, opening mouth showing teeth and/or softly biting the other otter, and sometimes vocalizing.
		Reacting while being groomed by turning its head towards the other otter and/or vocalizing.
**Affiliative**	**Social grooming.**	Both otters licking and/or scratching each other with their paws.
		Licking or scratching the other otter with paws.
		Being licked or scratched by another otter with its paws.
	**Social playing.** Playing with another otter either in or out of the water.	Swimming, walking or running one after another or together in an energetic manner and interacting physically with their mouths, paws and body contact with occasional soft bites.
	**Mating behavior.**	Swimming together with another otter, male gently biting female’s neck region, pre-copulatory behavior.
		Copulation.
	**Interacting/remaining close.** Interacting with either another otter or a person.	Interacting with otter from adjacent enclosure, either by touching each other’s paws, sniffing each other or trying to reach each other.
		Licking, scratching or biting its own fur while close to another otter or a human.
		Physically interacting with people (with otter’s arms reaching outside of the enclosure) through the fence.
**Soliciting/begging**	**Begging for food.**	Approaching and looking at another otter that was eating fish, walking or swimming towards it, trying to get its food
		Watching/observing another otter that is eating food, interested in the it.
	**Soliciting interaction.** Attempting to interact with either another otter or people.	Trying to approach and interact with another otter from the same enclosure by running or walking towards it.
		Trying to approach and interact with another otter from adjacent enclosure by running or walking towards it.
		Body positioned towards and gazing in direction to the other otter, moving in front of it to attract its attention.
		Trying to interact with people through the fence by vocalizing and sticking forelimbs through the fence while looking at people.
**Interested/observing/ moving towards it**	**Interested/observing/ moving towards it.** Attending to another otter or people.	Observing and paying attention to another otter.
		Observing another otter and moving its head/neck/body trying to get close to it.
		Climbing up the fence to look inside other otters’ enclosure.
		Running towards people.
		Walking towards people.
		Observing and paying attention to people.
**Environment interaction**	**Environmental interaction with excitement.** Interested and excited about the environment outside of their enclosure.	Running from one side to the other of the enclosure (usually pre feeding) or climbing up and down the fence.
		Run in direction to the fence.
		Climbing up the fence to look outside of their enclosures.
		Watching the environment outside of their enclosure in an excited manner, either rapidly moving eye gaze, head and neck movements, or standing up in their hind legs.
		Run in direction of the pool.
	**Environmental interaction with low excitement.** Interested in the environment outside of their enclosure.	Walking in direction of the fence.
		Watching the environment outside of their enclosure.
**Feeding**	**Feeding.** Waiting for food or walking away with food in an excited manner during feeding time.	Moving either head, body or eye gaze in a rapid manner while waiting for food to come out of the feeding tube or by the fence.
		Running in an exciting manner to obtain food.
		Walking rapidly with food in its mouth, just after receiving it.

### Behavioral context and structure of the vocalizations

We used the call occurrence data, which was collected regularly over eight months during the first field season, to evaluate the frequency of uttered calls (Table 2, S4 Table). We did not use the audio recorded data to evaluate call occurrence rate, since audio data collection was focused during a short period of time and the calls used for the audio analysis were selected for their sound quality, not emission rate, and thus do not accurately represent the total calls produced. Table 2 contains information of emission rate per call type and per sex only as descriptive statistics, there was no statistical test assessing differences in emission between call types and sex.

**Table 2:** Emission rate, mean and standard deviation (SD), per call type and sex.

Call type	Emission rate (calls/hour)					
	Per call type		Per call type for females		Per call type for males	
	Mean	SD	Mean	SD	Mean	SD
**Chirp**	3.9	3.3	2.4	3.3	4.8	3.1
**Squeak**	4	3.4	4.4	3.7	2	1
**Chuckle**	8.2	16	5	4.7	11.1	21.4
**Growl**	2.5	2.1	2.7	2.2	2.6	2.2
**Hah**	5	8.2	6.8	11.2	3.5	4.1
**Scream**	5.7	9.1	7.8	11.5	3.4	4.0

The frequency of uttered call types and their emission rate differed somewhat between males and females (Tables 2 and 3, S4 Table). The females in this study frequently uttered screams in an aggressive context, while males produced more chirps and chuckles in an affiliative context. However, this difference may reflect captive conditions and enforced proximity (see Discussion).

**Table 3:** Means, standard deviations (SD) and ranges for 12 (Tonal calls) and 4 (NLP calls) acoustic parameters of otters’ calls.

Tonal vs. irregular calls	Call Type (sample size)	Acoustic property	Mean	SD	Range
Tonal	Chirp (217)	Mean fundamental Frequency (f_0_) (Hz)	919.98	106.01	(621.24–1249.19)
		Minimum f_0_ (Hz)	646.57	108.08	(479.48–1027.37)
		Maximum f_0_ (Hz)	1147.40	126.67	(828.41–1410.41)
		Dominant Frequency (Hz)	1029.61	388.64	(550–2350)
		Standard deviation of f_0_ (Hz)	154.52	52.17	(41.40–286.08)
		Initial f_0_ (Hz)	914.02	169.13	(589.39–1303.59)
		Mid-point f_0_ (Hz)	1019.12	165.05	(577.91–1324.24)
		End f_0_ (Hz)	972.56	167.74	(594.72–1408.45)
		Slope First Half (Hz)	1621.41	10387.81	(-46381.54–30818.24)
		Slope Second Half (Hz)	-7020.44	7479.46	(-36257.84–17430.16)
		Duration (Sec)	0.08	0.08	(0.02–0.60)
		F_0_ Range (Hz)	500.82	141.93	(184.54–858.53)
	Squeak (9)	Mean f_0_ (Hz)	545.24	181.61	(334.91–772.21)
		Minimum f_0_ (Hz)	479.28	158.15	(302.04–699.55)
		Maximum f_0_ (Hz)	614.91	212.43	(349.63–835.90)
		Dominant Frequency (Hz)	690	114.02	(550–850)
		Standard deviation of f_0_ (Hz)	37.76	19.50	(17.88–59.42)
		Initial f_0_ (Hz)	540.38	179.78	(302.04–787.84)
		Mid-point f_0_ (Hz)	542.73	167.65	(345.62–771.68)
		End f_0_ (Hz)	511.81	178.13	(305.65–704.57)
		Slope First Half (Hz)	121.96	2858.64	(-4070.55–3631.36)
		Slope Second Half (Hz)	-3024.12	2763.12	(-4995.78–1780.92)
		Duration (Sec)	0.04	0.02	(0.02–0.06)
		F_0_ Range (Hz)	135.62	83.28	(47.58–235.40)
	Chuckle (62)	Mean f_0_ (Hz)	196.42	16.91	(174.35–229.92)
		Minimum f_0_ (Hz)	153.49	10.27	(136.55–182.44)
		Maximum f_0_ (Hz)	252.45	31.97	(204.24–301.41)
		Dominant Frequency (Hz)	577.78	196.46	(350–1050)
		Standard deviation of f_0_ (Hz)	27.26	12.59	(10.18–56.82)
		Initial f_0_ (Hz)	208.51	38.09	(162.77–291.56)
		Mid-point f_0_ (Hz)	197.95	33.04	(151.02–259.21)
		End f_0_ (Hz)	193.86	31.56	(151.12–270.29)
		Slope First Half (Hz)	-298.63	811.03	(-2161.74–842.42)
		Slope Second Half (Hz)	215.01	702.11	(-722.95–2348.72)
		Duration (Sec)	0.12	0.03	(0.09–0.18)
		F_0_ Range (Hz)	98.96	37.11	(36.77–162.96)
	Growl (4)	Mean f_0_ (Hz)	118.88	8.84	(106.88–127.62)
		Minimum f_0_ (Hz)	102.94	14.71	(88.71–122.43)
		Maximum f_0_ (Hz)	131.19	3.72	(126.02–134.57)
		Dominant Frequency (Hz)	975.00	556.03	(350–1650)
		Standard deviation of f_0_ (Hz)	7.01	3.62	(2.77–11.31)
		Initial f_0_ (Hz)	113.18	19.18	(88.75–132.77)
		Mid-point f_0_ (Hz)	121	9.55	(108.40–131.58)
		End f_0_ (Hz)	120.33	9.89	(108.06–129.01)
		Slope First Half (Hz)	-2.08	85.76	(-121.98–76.99)
		Slope Second Half (Hz)	-13.33	26.33	(-50.35–11.29)
		Duration (Sec)	0.44	0.33	(0.19–0.88)
		F_0_ Range (Hz)	28.26	12.49	(12.14–42.43)
Irregular	Hah (112)	Dominant Frequency (Hz)	1385.06	403.53	(750–3450)
		Duration (Sec)	0.50	0.11	(0.3–0.9)
		Tonal duration (Sec)	0	0	0
		NLP duration (Sec)	0.50	0.11	(0.3–0.9)
	Scream (94)	Dominant Frequency (Hz)	1700	1071.90	(550–4950)
		Duration (Sec)	0.50	0.45	(0.08–2.57)
		Tonal duration (Sec)	0.27	0.27	(0–1.38)
		NLP duration (Sec)	0.24	0.42	(0–2.57)

#### Chirp

The chirp is a harmonic sound with a high fundamental frequency with a mean f_0_ of 920 Hz (± SD 106 Hz) and dominant frequency of 1030 Hz (± SD 389 Hz). It is of short duration, 0.1 (± SD 0.08) sec., with its basic characteristics consistent across and within calls (Fig 1A). In the frequency domain it has an arch-shaped contour with high modulation, with a fundamental frequency that increases and decreases abruptly, thus exhibiting a high f_0_ range (mean 501 Hz, ± SD 142 Hz). The chirp is a contact call recorded in an affiliative context. It was emitted mainly when soliciting interaction from humans, or interacting with caretakers and the researcher, but was also sometimes directed to other otters. Chirps also occurred during social play and when first observing and later moving towards humans or other otters. It was uttered 3.9 times per hour on average, and was produced more by males than females (Table 2).

#### Squeak

The squeak is a low amplitude sound with an average fundamental frequency of 545 Hz (± SD 182 Hz) and a dominant frequency of 690 Hz (± SD 114 Hz) (Fig 1B). It is structurally similar to the chirp but has a lower fundamental frequency and is of lower perceived loudness. Squeaks were emitted during close social contact, mainly during social grooming and occasionally during social play, predominantly directed to otters from the same enclosure or secondarily emitted towards humans with whom the otter interacted closely (S4 Table). The individuals produced on average four squeaks per hour, and the females uttered more squeaks than males (Table 2).

#### Chuckle

The chuckle has a low fundamental frequency, 196 (± SD 17) Hz on average, and is a harmonic sound that is predominantly uttered in sequences and can be uttered in alternation with chirp calls (Fig 1C). It is a pulsating sound with an average of 3.6 (±SD 0.8) sub-vocalizations in sequence, and an inter-segment interval of 95 (±SD 19) milliseconds. It is the vocalization with the lowest dominant frequency (mean 578 ± SD 196 Hz) of all six calls we analyzed, with an almost constant fundamental frequency during its short duration of 0.12 (± SD 0.03) sec. The chuckle was emitted during soliciting or begging behavior, and mostly directed at humans, specifically when attempting to attract human attention or trying to reach and interact with humans. Chuckles were also uttered towards other otters when trying to interact and approach them in a affiliative social context, or when begging for food (S4 Table). The chuckle is one of the two calls with the highest emission rate (8.2 calls/hour) we observed, and it was produced more by males than females (Table 2).

#### Growl

The otter growl is a typical carnivore growl, and is tonal with a low and essentially constant pitch. The growl has the lowest average fundamental frequency of the six call types—119 Hz (± SD 8 Hz)—and the lowest minimum f_0_ of 102 Hz (± SD 15 Hz). It is also the most constant in fundamental frequency, with the lowest frequency range of 28 Hz (± SD 12 Hz) (Fig 1E). The growl was the most stereotyped call with its vocal characteristics consistent from call to call. It is a harmonic call with stacked harmonic bands. Despite its low f_0_, it has a high bandwidth and a high dominant frequency (maximum energy) at 975 (± SD 556) Hz. Growls are produced in threatening and/or warning contexts, with the caller protecting its own food or more generally indicating discontent with another otter’s proximity (S4 Table). The growl had the lowest emission rate (mean of 2.5 calls/hour) among all call types observed and was uttered with similar frequency by males and females (Table 2).

#### Hah

The “hah” call involves a forcible exhalation of air, lacking a clear fundamental frequency in the spectrogram; it is a broadband, noisy sound with a dominant frequency mean at 1385 (± SD 404) Hz (Fig 1D). Hahs are typically uttered repetitively in sequences. Hah calls were emitted mainly in alert, inquisitive contexts when something novel appeared in the caller’s environment. The call was produced while adopting an alert body posture, with the caller either erect and standing up on its hind legs, or with a stretched neck facing directed to the source of novelty. Hahs were associated with excitement and emitted on average five times per hour (Table 2), and often call emission intensified just before feeding time, when otters heard or saw their food source approaching. Hahs were also produced when otters were looking outside or running from one side to the other of the enclosure, when climbing up and down the enclosure’s fence, while walking with fish in their mouths, just after receiving fish, or when observing human activities (S4 Table).

#### Scream

Screams are uttered in an agonistic social context. Screams (Fig 1F) can be divided into two sub-call types, short-screams and long-screams that are acoustically similar with gradations between them. Screams have the highest dominant frequency of 1700 (± SD 1071) Hz compared to the other call types, and it is aurally perceived as the loudest call (Table 3). Screams have high frequency modulation and are often composed of both harmonic and non-harmonic segments. Screams vary considerably from call to call as shown by the standard deviation of acoustic parameters (Table 3). Screams have one of the highest emission rates (mean 5.7 ± SD 9.1 calls/hour) of all the vocalizations, and it was produced mainly by females and directed towards males (Table 2, S4 Table). Screams vary from a brief sound (short-scream) to a long sound (long-scream), their duration varying from 0.08 to 2.57 seconds.

**Fig 2:**
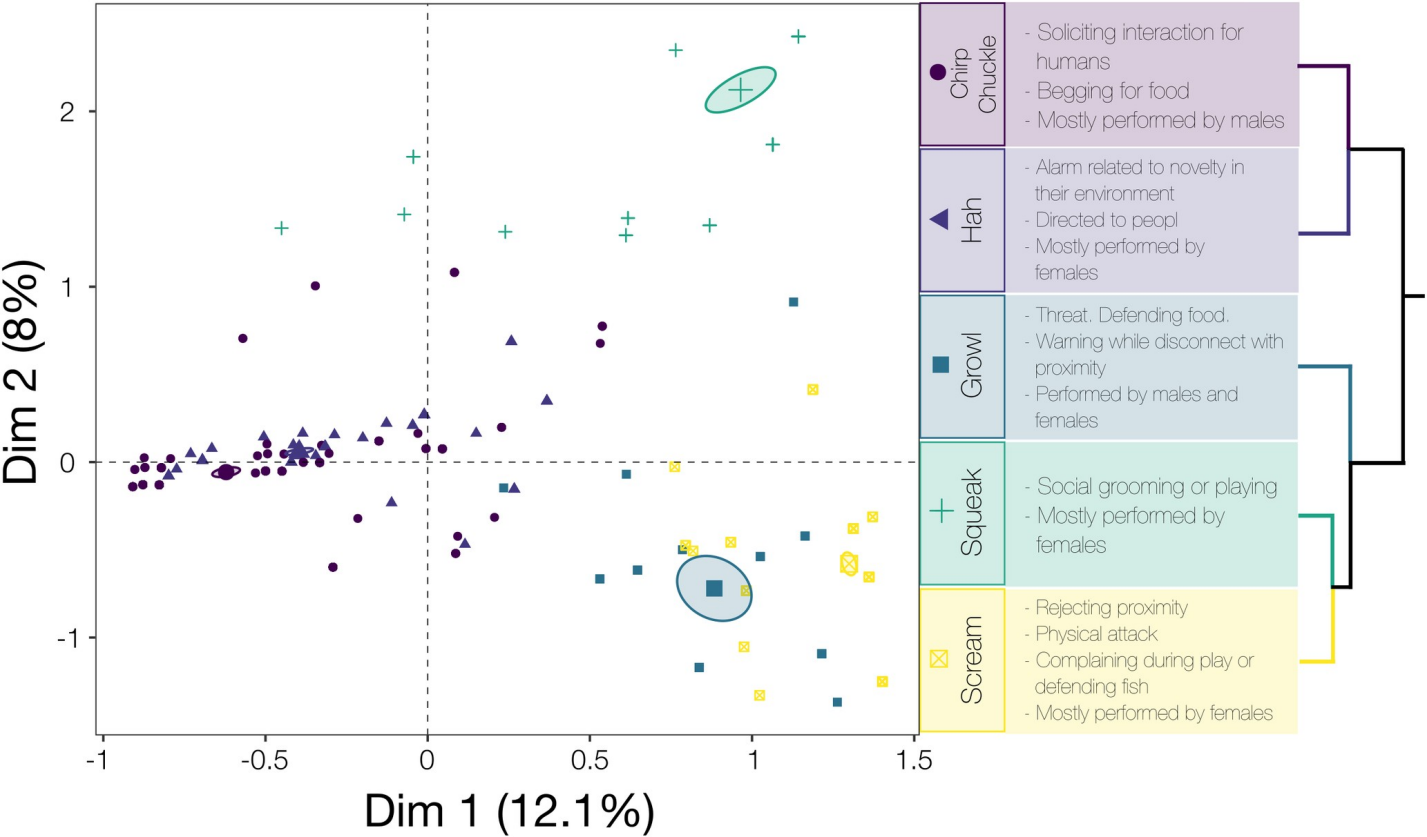
Biplot of multiple correspondence analysis with hierarchical clustering summarizing the association between call types, behavioral contexts and sexes. Larger symbols represent centroids, and ellipses represent 95% confidence intervals.

Short screams were produced when otters were unwillingly touched or approached by another individual including during social playing, often accompanied by the caller adopting a threatening face and body posture, and rejecting interaction. It occasionally progressed to the long scream when the calling individual was repeatedly approached (Fig 2, S4 Table). The long scream was uttered in a physical aggression context. The signaler uttered the sound while displaying physical threat postures and/or attack. It was uttered when otters rejected social interaction, during physical threat and attack.

### Association between call types and behavioral context

Five clusters were derived from the Multiple Correspondence Analysis (MCA) and subsequent hierarchical cluster analysis. Cluster 1 was characterized by males soliciting interaction from humans when performing the “chirp” and “chuckle” call types (Fig 2; S4 Table). Cluster 2 was characterized by females performing the “Hah” call type to people or showing alarm to novelty in their environment (Fig 2; S4 Table). Cluster 3 was characterized mainly by the emission of the “Growl” call type, by males and females in similar proportion, when defending food and/or warning during overly close proximity (Fig 2; S4 Table). Cluster 4 was characterized by females performing the “Squeak” call type in social grooming or play contexts (Fig 2; S4 Table). Finally, cluster 5 was characterized by females performing the “scream” call type when rejecting proximity, complaining during play or while defending fish and defending themselves from physical attack (Fig 2; S4 Table).

### Call type classification based on acoustic data

**Fig 3:**
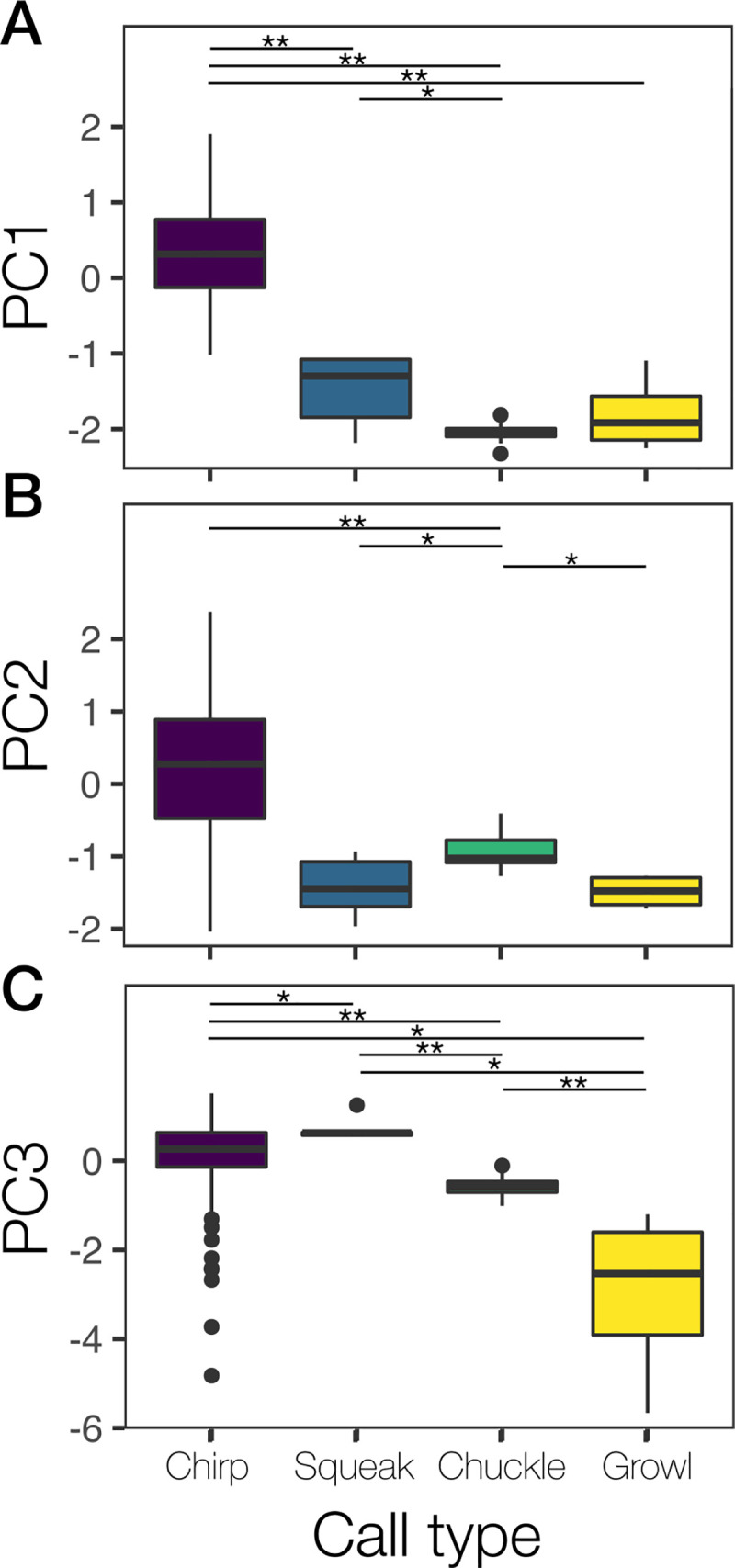
Box plot showing the difference between call types in their acoustic parameters represented by the first (A; explained variance = 59%), second (B; explained variance = 21%) and third (C; explained variance = 20%) principal components for Tonal calls. *P<0.05; **P<0.001.

The PCA for tonal calls generated three factors with eigen values greater than one. The first principal component (PC1) explained 59% of the total variance and represented positively most of the acoustic parameters (S4 Table). Pairwise comparisons showed significant differences in the PC1 between the “chirp” call type and the others (Wilcoxon rank sum test: all p-values <0.001; Fig 3A; S5 Table), as well as between the “squeak” and the “chuckle” call types (Wilcoxon rank sum test: p-value = 0.03; Fig 3A; S5 Table). The second principal component (PC2) explained 21% of the total variance and represented positively the dominant frequency and the rate of change of f_0_ in the second half (S4 Table). Pairwise comparisons showed significant differences in the PC2 only between the “chuckle” call type and the others (Wilcoxon rank sum test: all p-values <0.05; Fig 3B; S5 Table). Finally, the third principal component (PC3) explained the remaining 20% of the total variance and represented negatively the duration of the call (S4 Table). Pairwise comparisons showed significant differences in the PC3 between all call types (Wilcoxon rank sum test: all p-values <0.05; Fig 3C; S5 Table).

**Fig 4:**
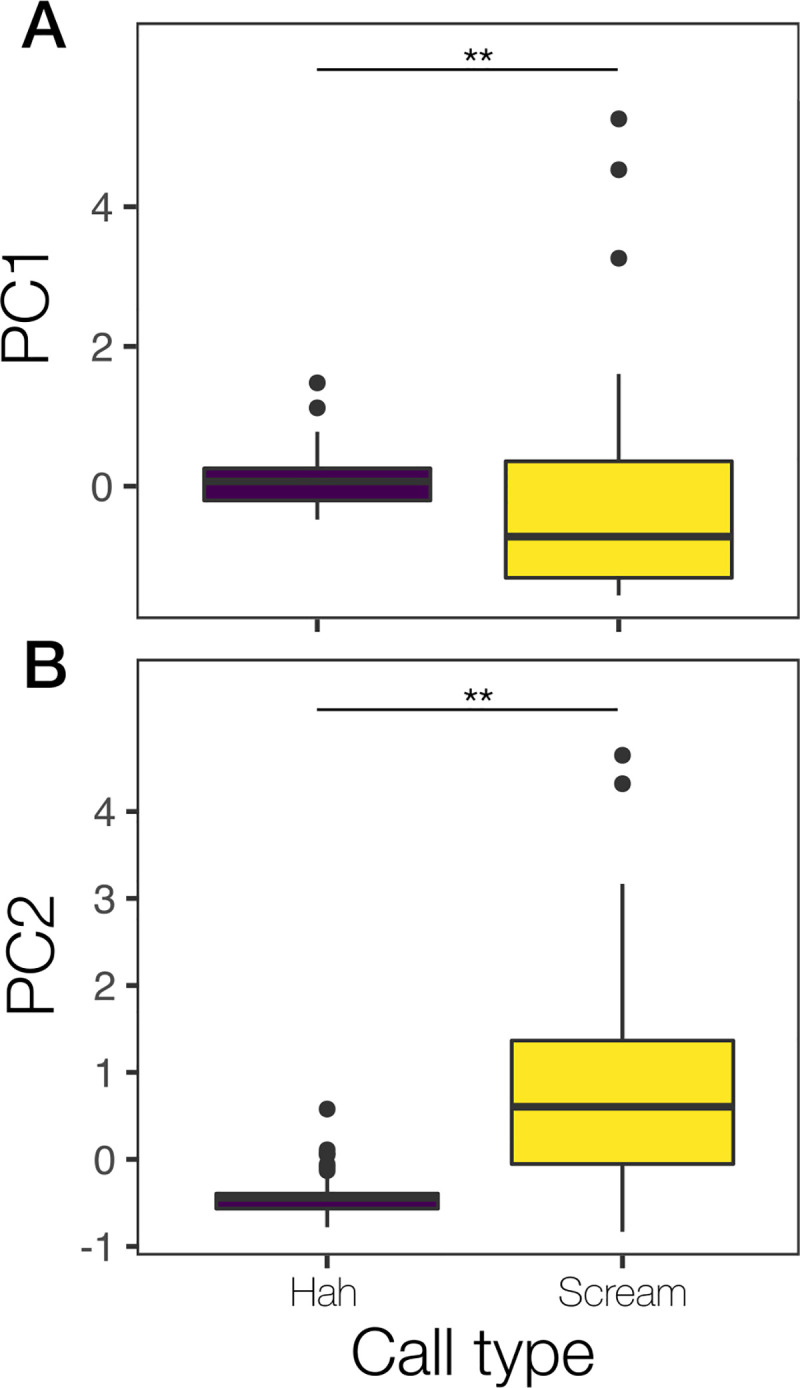
Box plot showing the difference between call types in their acoustic parameters represented by the first (A; explained variance = 56%) and second (B; explained variance = 44%) principal components for NLP calls. **P<0.001.

Similarly, the PCA for irregular calls generated two factors with eigen values greater than one (S6 Table). The first principal component (PC1), which explained 56% of the total variance, represented positively the duration of the call and NLP duration (S6 Table). The second principal component (PC2) explained the remaining 44% of the total variance and represented positively the dominant frequency and tonal duration (S6 Table). The “Hah” and the “scream” call types were significantly different in both, PC1 (Kruskal-Wallis test: X^2^ = 12, df = 1, p-value <0.001; Fig 4A; S7 Table) and PC2 (Kruskal-Wallis test: X^2^ = 42, df = 1, p-value <0.001; Fig 4B; S7 Table). Our initial assumptions were of seven call types, chirp, squeak, chuckle, hah, short-scream, long-scream and growl, based on preliminary auditory categorization. Subsequently based on these results we classified all calls into only six call types, the chirp, squeak, chuckle, hah, growl, and scream (collapsing short-scream and long-scream into one graded category).

## Discussion

In this study we described the vocal repertoire of the neotropical otter in captivity, providing the first acoustically detailed description of neotropical otter vocal communication. The vocal repertoire includes both tonal calls and noisy vocalizations containing nonlinear phenomena, with tonal calls including fundamental frequency ranges from 90Hz to 2500Hz. Our analyses revealed six acoustically distinct call types which were classified based on acoustic characteristics, visual inspection of spectrogram and how they are perceived aurally by human observers.

According to the HCPC analysis, chuckle and chirp were grouped in cluster 1, represented a group of affiliative and orientation behaviors, which included attending to another otter or people, begging for food, soliciting interaction from another otter or people, and interested in the environment outside of their enclosure (Fig 2, S4 Table). However, is worth noting that chuckle was performed when begging for food while chirp was not. There were significant differences in the PC1, PC2 and PC3 between chirp and chuckle, thus, they are acoustically distinct call types (with chuckles pulsatile and longer in duration relative to the short chirps). Their spectrograms are visually different as well as how there are perceived aurally. Thus, chirp and chuckle were defined as distinct call types.

There is significant difference in the PC3 between growl and all the other call types. PC3 is mostly represented by call duration (see S5 Table). Thus, growl is distinct from the other call types by its call duration. PC2 revealed no significant difference between growl, chirp and squeak and PC1 no difference between growl and squeak. The fundamental frequency related parameters of growl, chirp and squeak are different, growl being characterized by a low mean f _0_ (119 Hz), chirp by a high mean f _0_ (920 Hz) and squeak by a mean f _0_ of 545 Hz (see Table 3). The sample size of growl (4) and squeak (9) are considerably low which could have potentially impacted the results of the PCA. Perhaps with a higher sample of growl and squeak we would have found significant differences between those two calls in the PC1 and PC2, which are represented by fundamental frequencies related parameters. Growl, squeak and chuckle behavioral context, call duration, visual aspect of spectrogram and how it is perceived aurally are distinct from one another. Therefore, we classified growl, squeak and chuckle as distinct call types.

Scream calls often contained nonlinear phenomena (NLP), in common with screams in many other mammalian species [12]. Scream calls typically included segments of NLP along with clear tonal “windows” where harmonics were visible in the spectrogram. The presence of NLP in mammal vocal communication systems may contribute to signaling arousal, motivation and status [14], and the relative presence of NLP and tonal segments in neotropical otter calls may indicate the level of arousal during call production, where higher NLP ratios might indicate higher arousal. This could be explored in future work where arousal level could be evaluated objectively.

### Call types and frequency of use

To calculate call occurrence rate (Table 2), we used data from our first data collection period, which was collected consistently over ten months, to evaluate the frequency of uttered calls. We did not include the audio recordings dataset, which was collected over a small window of three weeks, and selected for only high-quality calls, because these data do not represent accurately the call occurrence rate.

The most common call type observed was the chuckle, followed by the hah. Both were associated with affiliative contexts, specifically begging and soliciting as well as showing curiosity about novel events or objects in their environment. The individuals in this study were frequently attentive to human activities and often altered their behavior depending on human actions. Chirps and squeaks were also commonly heard, also in affiliative contexts, with chirps associated most frequently with solicitation of food or contact, and squeaks occurring during close-contact grooming and playing (Fig 2, S4 Table).

Screams were also frequently recorded, and clearly associated with agonistic (defensive or aggressive) social interactions. Females uttered more scream calls, compared to males, often screaming while rejecting solicited contact and interaction from their paired male mates, as well as from otters in the adjacent enclosures. The least-used call type was the growl, also uttered during aggressive and defensive situations such as defending food from an approaching conspecific (Fig 2, S3 and S4 Tables).

However, these data on call frequency are based on captive otters and must be treated with caution. Neotropical otters are considered to be solitary (other than mothers with pups) and are thought to rarely have physical contact or close encounters with other otters in the wild, where individuals use chemical communication to avoid agonistic encounters and physical conflict [26]. The individuals from this study were housed in pairs, which might have altered their natural behavior and the use of their vocal communication (S1 Table). Females often rejected interaction from their paired males, and from otters in the adjacent enclosures. Females uttered more scream calls associated with aggressive social behavior, compared to males (Table 2, S4 Table). This could differ substantially in free-living animals that are not forced to share an overlapping territory, and have more possibilities to avoid close contact and physical interaction. Thus, our findings cannot be directly extrapolated to wild populations, and further studies are needed in the wild for a broader understanding of the frequency of calling in the neotropical otter vocal communication system. This study also did not investigate nocturnal activities, agonistic encounters related to territory defense, underwater calls, mating calls, or calls from cubs and juveniles (e.g. while nursing or soliciting care). However, these and other social/environmental context could potentially be relevant to the neotropical river otter vocal communication system. Our summary here must thus be considered preliminary, but forms a basis for further investigations on free-living animals in a wider variety of social and ecological contexts.

### Comparison to other otter species’ vocalizations

**Fig 5:**
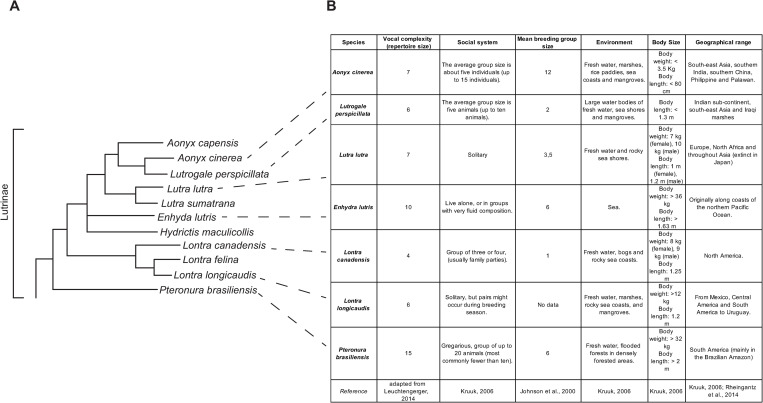
General information about otter species. (A) Phylogenetic tree (adapted from Koepfli et al. 2008 [10]); (B) Overview of otter species whose vocal repertoires have been studied.

Because all otters are closely related, comprising the carnivoran subfamily Lutrinae [41], and share similar ecological habits and body form, one might expect the 13 extant otter species to have similar vocalizations and vocal repertoires [42] (See Fig 5). On the other hand, otters vary considerably in their social system, from the mostly solitary neotropical otter studied here to highly gregarious otters that live in family groups with long-term male/female bonds (the giant otter *Pteronura*). Previous work in other mammal groups (particularly primates) suggests that vocal repertoire evolves in concert with social complexity, such that species with more complex social systems have more complex vocal repertoires [43]. From this viewpoint, we might expect otter species to vary considerably in their vocal repertoire. It is thus interesting to compare the repertoire of the neotropical otter published data from other species. Unfortunately, although vocalizations are reported for eight otter species, published descriptions of the vocal repertoire exist for only six species [1,2,6–8,17,18,42,44], limiting the degree to which current data can clarify vocal evolution within the Lutrinae subfamily.

Several call types found in the neotropical otter are shared with other otter species, prominently including chirps, hahs, and growls. The chirp call appears to be a common call type among all otter species, and chirps or similar vocalizations are described as part of the vocal repertoire of six other otter species: giant otters, river otters, sea otters, Asian small-clawed otters, Eurasian otters and smooth-coated otters [1,2,6–8,17,18,42]. In river otters, chirps occurred mostly during investigation and never in an agonistic context [7]. Giant otter “contact calls” [17] and adult calls [2] acoustically resemble the chirp in the neotropical otters. In short-clawed otters, the arched tonal “U3 call” analogous to the chirp is also uttered during affiliative interaction and exploration [1]. Eurasian otters use “whistles” comparable to chirps, as both a close and distant contact call [8]. Finally, sea otters produce “squeaks” that are acoustically comparable to chirps [18]. Thus, among all the shared call types across otter species, the chirp seems to be homologous among all the studied otter species [1,2,6–8,17,18,42] which belong to all three primary phylogenetic clades among the Lutrinae. Its acoustic structure and behavioral context seems to be consistent across species, suggesting that chirps may also have been present in the vocal repertoire of the common ancestor of all extant otter species.

This widely used chirp might represent an environmental adaptation in otters. Calls produced at the water surface are prone to low-frequency sound attenuation, due to refraction and subtractive interference from reflections from the water surface, and can thus suffer substantial excess attenuation compared to high pitch sounds with shorter wavelengths [45]. Chirp calls are loud calls with a high fundamental frequency, and so should suffer less than lower pitch sounds from such “ground effects” when produced at the water surface by a swimming otter. Because otters typically vocalize at the water surface, this suggests that chirps may represent an adaptation to the aquatic environment in otters. Specifically, we hypothesize that the high fundamental frequency of otter chirps may be an adaptation to travel more efficiently and over longer distances on water surface (suffering less refractive attenuation).

Interestingly chirps in giant otters have higher fundamental frequencies than those of the much smaller neotropical otter. Fundamental frequency is often supposed to obey general allometric principles in which larger animals produce lower f_0_ and smaller animals higher f_0_ [46,47]. Because giant otters are considerably larger than river otters and neotropical otters (*Lontra canadensis*, *Lontra longicaudis*), they violate this prediction. However, larynx size and body size can vary independently of one another [48]. Because allometry between body size and formant frequencies often seems to be more reliable than that with fundamental frequencies [49–51], it would be interesting to know how formant frequencies vary in giant otters relative to those in New World river otters.

The ‘hah’ or a similar call is found to be present in the vocal repertoire of giant otters, Asian small-clawed otters, Eurasian otters and river otters, also in a context of environmental novelty and alarm (Almonte [7] and Gnoli and Prigioni [8] call it the “blow” in river otters and Eurasian otters, while Duplaix [42] terms it “hah” and Lemasson et al. [5] name it “U1”). The acoustic characteristics of the ‘hah’ among species are similar: it is an atonal call involving a forceful exhalation of air, with a broad frequency range. It is also similar to the hiss sound commonly given in aggressive and fearful contexts by sea otters [18] and various other mustelids [52–54] and carnivores (e.g. cats [55]).

Regarding screams, Duplaix [56] reports that “wavering screams” are a relatively frequently emitted vocal type in giant otters, also used in contexts of threat or frustration. Although screams have not been reported in other otter vocal repertoires, this may reflect observation conditions, because, as mentioned above, keeping the naturally solitary neotropical otters in pair-housed conditions may have increased the frequency of agonistic interactions observed in our study population. Thus, we would not be surprised if screams are also present in other, more sociable, otters but are less frequently observed due to the rarity of agonistic encounters.

Scream calls in giant otters can be uttered simultaneously by group members to produce a group territorial “chorus” that encodes information on group identity [4]. Giant otters’ screams are loud, long-range vocalization with low dominant frequency, and a high rate of amplitude modulation; they are rough sounds reported to travel long distances. Neotropical otters’ screams are also loud with high amplitude modulation, however we did not observe screams in territorial choruses like those of the social giant otter. Nevertheless, screams might be used by wild population in agonistic encounters, in a similar way to giant otters, to achieve mutual avoidance of different otters sharing the same territory.

The growl is a common and familiar call among carnivores in general, and has also been described in the vocal repertoire of the giant otter [56]. The acoustic characteristics and behavioral contexts of growls seem to be highly similar among neotropical otters and giant otters. This low frequency, harmonic and pulsed sound is emitted in a threat and defensive context.

Finally, a friendly pulsed vocal type, termed the "twitter" or "chuckle," appears to be shared by North American river otters, neotropical otters and Eurasian otters [7,8]. Asian small-clawed otters, smooth-coated otters, sea otters and giant otters appear to use an acoustically distinct call type, the “coo," to subserve friendly contact [1,2,18,42].

Despite multiple call types being shared across otter species, giant otters seem to have the most distinct vocal communication from all the otter species described so far: they have the most complex vocal repertoire, and their vocalization rate seems to be the highest among those otter species studied. Giant otters represent a phylogenetically basal clade, separate from all otter species, within the Lutrinae [57]. Thus, the differences in their vocal communication compared to other otter species may reflect the greater phylogenetic distance between giant otters and other otters (Fig 5). In contrast, closely related species may share more aspects of vocal communication. River otters *L*. *canadensis* are often solitary, but can be much more gregarious than neotropical otters [26]. Even given this difference in social systems, the river otter seems to have the most homologous vocal repertoire of neotropical otters. This might reflect their phylogenetic proximity, being from the same genus and primary linage, New World river otters, within the Lutrinae [57].

However, at a finer level, even shared call types among otter species have different acoustic parameters in different species. These differences in acoustics may be correlated with body size, social structure, ecology or phylogeny. To accurately evaluate the similarities and differences across otter vocal repertoires, there is a need for a phylogenetically controlled study in which consistent acoustic parameters are extracted from shared call types across otter species, and explicitly evaluated relative to phylogenetic similarities.

Turning to repertoire size, there is a large variation of reported repertoire size in otters, ranging from four call types (river otter) to 15 call types (giant otters) within the Lutrinae [2,7]. McComb and Semple [43] have suggested that primate species with larger group sizes have more complex vocal repertoire, and Leuchtenberger et al. [2] found a positive correlation between vocal complexity and sociability in mustelids, including otters, supporting the social intelligence hypothesis of Freeberg et al. [58]. However, in social mongoose species there is no correlation between group size and vocal repertoire size, their vocal complexity is being better explained by their social organization and the ecology of the species [59].

The neotropical otter is a mostly solitary species, and the vocal repertoire of six call types described here may indicate a comparable vocal complexity to the mostly solitary Eurasian otters, with a vocal repertoire of seven call types [8], and a less complex vocal system than the highly social giant otters and sea otters, with vocal repertoires of, respectively, 15 and 10 call types [2,18]. Neotropical river otters thus may be consistent with the putative correlation between vocal complexity and sociability. However, multiple factors including different housing and recording conditions, along with different analysis methods, for different species make it difficult, given our present state of knowledge, to reach any firm conclusions about vocal repertoire size, and published repertoire sizes might also be misleading. Because only some vocal repertoire studies included descriptions of infant and juvenile calls, we should perhaps base the comparison of vocal repertoire size across otter species only on the size of adult vocal repertoire.

Furthermore, according to Mumm & Knörnschild [17], group size alone is an inadequate measure for social complexity, and other aspects of social behavior should be considered. To represent more accurately a measure for social complexity in otters we should include other social living aspects that take account of both the quantity (group size) and quality of relationships within social groups other than just group size. McComb [43] uses two indices of social bonding in primates: group size and time spent grooming, to assess whether evolutionary changes in vocal repertoire size are linked to social complexity. Some aspects, for example time spent in close proximity or time spent playing, could also provide a measure of the quality of social bonding in otters. We thus conclude that it is premature, given the current state of knowledge, to attempt to use social complexity to explain the putative variation of vocal repertoire size among different otters species.

## Conclusions

The neotropical river otter’s vocal communication system, as documented in this captive study, includes at least six distinct call types, several of which are highly similar to those recorded in other otter species. We found no evidence for any unusual call types specific to this species. Despite considerable variation in the reported repertoire size of different otter species, it remains unclear to what extent these numerical differences reflect true biological differences in species’ repertoire size as opposed to stemming from methodological and/or housing differences. The great variation in otter social systems (from the essentially solitary neotropical otter to highly gregarious giant and sea otters) makes this an issue well worth pursuing in future research, to test hypotheses about the relationship between social complexity and vocal complexity. Similarly, it remains unknown whether otters show specific changes in vocal anatomy that may reflect adaptations to aquatic life, as seen in many other aquatic mammals. Thus, further comparative studies on the vocal communication of both solitary and social otter species might reveal aspects of the evolution of the vocal communication system in the subfamily Lutrinae that are relevant to larger issues in the evolution of communication.

Finally, although our results stem from captive animals, and thus may not fully reflect the vocal repertoire or call usage of this species, our results have potential for use in captive otter management. Improved knowledge of apparent correlations between call type, and specific call parameters, to arousal state of the caller might potentially be used to improve neotropical otters’ welfare in captivity, e.g. by remotely and constantly monitoring arousal or stress levels via vocal measurements. In conclusion, the findings reported here reveal fundamental aspects of the vocal repertoire of the neotropical otter, a solitary otter species, and thus contribute more broadly to the understanding of the vocal communication systems of the Lutrinae, an understudied semi-aquatic carnivore subfamily.

## Supporting information

S1 Table Information on housing condition of the studied otters. (DOCX)

S2 Table Input parameters for audio analysis. Set of input parameters per call types used in Praat to extract sound parameters.
(DOCX)

S3 Table Multiple correspondence analysis showing the dimensions with cumulative proportion of explained variance greater than 0.8 and the contribution of the call types and the behaviors in every dimension. (DOCX)

S4 Table Within-cluster distribution of behaviors, call types and sex. Mod. Cla represents the percentage of individuals that performed each behavior.
(DOCX)

S5 Table Principal components analysis showing principal components with cumulative proportion of explained variance greater than 0.8 and loadings matrix for 12 acoustic parameters in Tonal calls of otters. (DOCX)

S6 Table Summarized results of Tukey multiple comparisons of means between acoustic parameters (PC1) of tonal call types. (DOCX)

S7 Table Principal Components Analysis showing principal components with cumulative proportion greater than 0.8 and loadings matrix for four acoustic parameters in NLP calls of otters. (DOCX)

S8 Table Summarized results of Tukey multiple comparisons of means between acoustic parameters (PC1) of NPL call types. (DOCX)

S1 Audio Chirp call. (WAV)

S2 Audio Chuckle call. (WAV)

S3 Audio Squeak call. (WAV)

S4 Audio Hah call. (WAV)

S5 Audio Growl call. (WAV)

S6 Audio Scream call. (WAV)
