# Supplementary material for: Airborne vocal communication in adult neotropical otters (Lontra longicaudis)
Source: PLoS One. 2021 May 26;16(5):e0251974. doi: 10.1371/journal.pone.0251974 (PMC8153427; doi:10.1371/journal.pone.0251974)
Supplement: S1 Table — (DOCX) [file pone.0251974.s001.docx]

**Table S1.** Information on housing condition of the studied otters.

| **Housing information** | **1st data collection** (September 2013 till December 2014) | **2nd data collection** (January 2017) |
| --- | --- | --- |
| **Number of individuals recorded** | 6 | 5 |
| **Identity of individuals (f=female; m=male)** | Iara (f), Bela (f), Pretinha (f), Tupi (m), Peri (m), Boni (m). | Iara (f), Bela (f), Tupi (m), Peri (m), Boni (m). *Pretinha died. |
| **Number of enclosures** | 3 | 3 |
| **Enclosure walls/edges** | See through wire fence. | Opaque walls with few see through glass. |
| **Enclosure I** | Habitants: Iara (f) and Tupi (m). | Habitants: Tupi (m). |
|  | Size: 7 meters (length) and 3 meters (width). | Size: Awkward shape, approximately 19 meters (length) and 6.5 meters (width). |
|  | Contact with other enclosures: visual, auditory and physical (limited) contact with enclosure II; auditory, visual (limited) with enclosure III. | Contact with other enclosures: visual (limited), auditory contact with enclosure II; auditory contact with enclosure III. |
|  | Structure: Outdoors area, ground had sandy areas but mostly covered with grass. With one swimming pool deep enough to provide diving area for the otters and there were two shelters. | Structure: Outdoors area, ground had sandy areas but mostly covered with grass. With two interconnected swimming pools deep enough to provide diving area for the otters and there were two shelters. |
| **Enclosure II** | Habitants: Bela (f) and Peri (m). | Habitants: Bela (f) and Peri (m). |
|  | Size: 7 meters (length) and 3 meters (width). | Size: Awkward shape, approximately 14 meters (length) and 8 meters (width). |
|  | Contact with other enclosures: visual, auditory and physical (limited) contact with enclosure I and III | Contact with other enclosures: visual (limited) and auditory contact with enclosure I and III. |
|  | Structure: Outdoors area, ground had sandy areas but mostly covered with grass. With one swimming pool deep enough to provide diving area for the otters and there were two shelters. | Structure: Outdoors area, ground had sandy areas but mostly covered with grass. With two swimming pools deep enough to provide diving area for the otters and there were two shelters. |
| **Enclosure III** | Habitants: Pretinha (f) and Boni (m). | Habitants: Iara (f) and Boni (m). |
|  | Size: 12 meters (length) and 6 meters (width). | Size: Awkward shape, approximately 12.5 meters (length) and 8 meters (width). |
|  | Contact with other enclosures: visual, auditory and physical (limited) contact with enclosure II; auditory, visual (limited) with enclosure I. | Contact with other enclosures: visual (limited), auditory contact with enclosure II; auditory with enclosure I. |
|  | Structure: Outdoors area, ground had sandy areas but mostly covered with grass. With one swimming pool deep enough to provide diving area for the otters and there were two shelters. | Structure: Outdoors area, ground had sandy areas but mostly covered with grass. With two swimming pools deep enough to provide diving area for the otters and there were two shelters. |
