# Supplementary material for: Airborne vocal communication in adult neotropical otters (Lontra longicaudis)
Source: PLoS One. 2021 May 26;16(5):e0251974. doi: 10.1371/journal.pone.0251974 (PMC8153427; doi:10.1371/journal.pone.0251974)
Supplement: S2 Table — Set of input parameters per call types used in Praat to extract sound parameters. (DOCX) [file pone.0251974.s002.docx]

**Table S2.** Input parameters for audio analysis. Set of input parameters per call types used in Praat to extract sound parameters.

| **Spectrogram and Pitch settings** | **Chirp** | **Squeak** | **Chuckle** | **Growl** | **Hah** | **Scream** |
| --- | --- | --- | --- | --- | --- | --- |
| **View range (Hz)** | 0-300 | 0-5000 | 0-7000 | 0-3000 | 0-7000 | 0-5000 |
| **Window length (s)** | 0.01 | 0.02 | 0.02 | 0.035 | 0.01 | 0.02 |
| **Dynamic range (dB)** | 65.0 | 65.0 | 65.0 | 65.0 | 65.0 | 65.0 |
| **Pitch range (Hz)** | 500-1450 | 400 - 900 | 150 - 300 | 80-180 | - | 700 - 3000 |
| **Max numb. Of candidates** | 15 | 15 | 15 | 15 | - | 15 |
| **Silence threshold** | 1 | 0.05 | 0.1/0.001 | 0.07 | - | 0.03/0.07 |
| **Voicing threshold** | 0.2 | 0.08 | 0.05 | 0.05 | - | 0.05 |
| **Octave cost** | 0.01 | 0.01 | 0.01 | 0.01 | - | 0.01 |
| **Octave jump cost** | 0.35 | 0.35 | 0.35 | 0.35 | - | 0.35 |
| **voiced/unvoiced cost** | 0.14 | 0.14 | 0.14 | 0.14 | - | 0.14 |
| **Filter pass low** | 500 | 300 | - | 300 | 700 | 500 |
| **Filter pass high** | 2400 | 2600 | 1500 | 2400 | 3500 | 7500/8000 |
