# Supplementary material for: Airborne vocal communication in adult neotropical otters (Lontra longicaudis)
Source: PLoS One. 2021 May 26;16(5):e0251974. doi: 10.1371/journal.pone.0251974 (PMC8153427; doi:10.1371/journal.pone.0251974)
Supplement: S3 Table — (DOCX) [file pone.0251974.s003.docx]

**Table S3.** Multiple Correspondence Analysis showing the dimensions with cumulative proportion of explained variance greater than 0.8 and the contribution of the call types and the behaviours in every dimension.

| **Variable** | | **Dimensions** | | | | | | | | | | | | | |
| --- | --- | --- | --- | --- | --- | --- | --- | --- | --- | --- | --- | --- | --- | --- | --- |
|  |  | **1** | **2** | **3** | **4** | **5** | **6** | **7** | **8** | **9** | **10** | **11** | **12** | **13** | **14** |
| **Sex** | **Female** | 6.59 | 0.28 | 2.84 | 0.27 | 0.31 | 0.38 | 0.40 | 2.57 | 0.54 | 0.53 | 0.12 | 0 | 0 | 0.13 |
|  | **Male** | 7.64 | 0.33 | 3.30 | 0.32 | 0.36 | 0.44 | 0.46 | 2.97 | 0.63 | 0.61 | 0.14 | 0 | 0 | 0.15 |
| **Directed to** | **Environment** | 0.07 | 0.01 | 0.17 | 0.01 | 0.01 | 15.20 | 12.80 | 5.54 | 12.80 | 0.03 | 0.03 | 0 | 0 | 0.01 |
|  | **Feeding object** | 0.03 | 0 | 0.64 | 0.02 | 0.09 | 0.02 | 0.04 | 0.38 | 0.16 | 31.30 | 14.50 | 0 | 0 | 51.70 |
|  | **Itself** | 0.01 | 0.42 | 0.98 | 0.10 | 24.70 | 7.36 | 15.30 | 0.85 | 0 | 0.02 | 0.04 | 0 | 0 | 0 |
|  | **Otter adjacent enclosure** | 3.05 | 6.91 | 2.44 | 0.04 | 13.20 | 3.89 | 7.83 | 5.77 | 0.44 | 0.04 | 0.03 | 0 | 0 | 0 |
|  | **Otter same enclosure** | 11.00 | 1.89 | 0.17 | 0.15 | 7.03 | 1.95 | 3.64 | 0.84 | 0.19 | 0.02 | 0.11 | 0 | 0 | 0.02 |
|  | **People** | 10.50 | 0.03 | 0.78 | 0.04 | 0.21 | 0.88 | 0.14 | 1.81 | 0.84 | 0 | 0.26 | 0 | 0 | 0.16 |
| **Call type**  **(# call occurrences)** | **Chirp (68)** | 1.17 | 0.10 | 3.89 | 0.13 | 0.28 | 20.10 | 10.70 | 0.42 | 7.26 | 0.01 | 0.01 | 0 | 0 | 0.02 |
|  | **Squeak (67)** | 2.87 | 33.30 | 1.52 | 0 | 0.94 | 2.36 | 1.42 | 0.01 | 0.10 | 0.15 | 0.10 | 0 | 0 | 0.01 |
|  | **Chuckle (404)** | 7.39 | 0.24 | 5.12 | 0.42 | 0.38 | 4.06 | 2.48 | 0.53 | 1.15 | 0.15 | 0.42 | 0 | 0 | 0.01 |
|  | **Growl (28)** | 1.09 | 2.03 | 1.02 | 43.70 | 0.92 | 0.23 | 0.48 | 0.13 | 0.01 | 0 | 0 | 0 | 0 | 0 |
|  | **Hah (197)** | 0.92 | 0.21 | 31.70 | 0.44 | 0.85 | 0.61 | 0.01 | 2.82 | 0 | 0.04 | 0.06 | 0 | 0 | 0.08 |
|  | **Scream (201)** | 16.50 | 7.55 | 0.02 | 4.10 | 0.42 | 0.29 | 0.54 | 0.88 | 0.02 | 0.34 | 0.19 | 0 | 0 | 0.02 |
| **Behaviours** | **Begging for food (16)** | 0 | 0.05 | 0.02 | 0.04 | 1.76 | 0.33 | 5.34 | 7.37 | 16.70 | 0.04 | 42.30 | 0.28 | 0.01 | 11.50 |
|  | **Defending from attack (4)** | 0.12 | 0.10 | 0.09 | 0.11 | 0.79 | 0.89 | 1.52 | 0.58 | 1.47 | 54.80 | 17.90 | 2.65 | 0.09 | 13.00 |
|  | **Defending its food (20)** | 0.58 | 0.61 | 0.36 | 44.20 | 0 | 0.01 | 0 | 0.19 | 0.00 | 0.10 | 0.04 | 0.16 | 1.18 | 0.09 |
|  | **Environment High (220)** | 2.63 | 0.02 | 23.00 | 0.01 | 2.73 | 0.49 | 1.59 | 2.03 | 1.19 | 1.54 | 1.73 | 0 | 0 | 4.22 |
|  | **Environment low (26)** | 0.85 | 0.02 | 0.92 | 0.06 | 0.18 | 7.93 | 5.88 | 6.91 | 23.10 | 0.18 | 0.27 | 0 | 0 | 1.72 |
|  | **Feeding (27)** | 0 | 0.24 | 5.54 | 0.41 | 0.44 | 4.38 | 2.22 | 29.90 | 3.20 | 5.59 | 14.90 | 2.82 | 0.14 | 8.44 |
|  | **Interacting or close by (11)** | 0.01 | 2.23 | 2.12 | 0.11 | 33.70 | 5.22 | 6.32 | 0.07 | 0.03 | 0 | 0.01 | 0 | 0 | 0 |
|  | **Interested at (137)** | 3.53 | 0.08 | 1.21 | 0.15 | 0.33 | 9.71 | 4.55 | 4.17 | 5.24 | 1.41 | 5.53 | 4.76 | 1.16 | 6.82 |
|  | **Mating (4)** | 0.37 | 0.04 | 0.01 | 0.25 | 0.45 | 0.32 | 0.70 | 1.05 | 0.21 | 0.03 | 0.09 | 19.10 | 73.10 | 0.01 |
|  | **Physical attack (74)** | 6.53 | 7.84 | 0.42 | 4.49 | 4.90 | 3.49 | 6.70 | 6.22 | 2.83 | 0 | 0.02 | 0.58 | 1.11 | 0 |
|  | **Reject interaction or proximity (131)** | 9.28 | 2.85 | 0.13 | 0.02 | 2.95 | 2.36 | 5.25 | 10.00 | 2.38 | 0.41 | 0.07 | 0.91 | 10.40 | 0.78 |
|  | **Social grooming (51)** | 2.85 | 29.40 | 0.95 | 0.10 | 0.09 | 4.07 | 2.64 | 0.01 | 0.97 | 0.67 | 0.16 | 5.23 | 0.98 | 0.01 |
|  | **Social play (23)** | 0.79 | 3.02 | 0.01 | 0.04 | 0.82 | 2.11 | 0.18 | 0.68 | 4.60 | 2.05 | 0.37 | 59.10 | 11.00 | 0.47 |
|  | **Soliciting Interaction (221)** | 3.62 | 0.13 | 10.60 | 0.31 | 1.19 | 0.91 | 0.87 | 5.28 | 13.90 | 0.03 | 0.64 | 4.44 | 0.86 | 0.67 |
| **Proportion of explained variance** | | 12.09 | 8.01 | 7.49 | 6.59 | 6.21 | 5.45 | 5.43 | 4.87 | 4.52 | 4.25 | 4.23 | 4.17 | 4.17 | 4.09 |
| **Cumulative proportion** | | 12.09 | 20.10 | 27.59 | 34.18 | 40.39 | 45.84 | 51.27 | 56.14 | 60.66 | 64.90 | 69.13 | 73.30 | 77.46 | 81.55 |
