# Supplementary material for: Airborne vocal communication in adult neotropical otters (Lontra longicaudis)
Source: PLoS One. 2021 May 26;16(5):e0251974. doi: 10.1371/journal.pone.0251974 (PMC8153427; doi:10.1371/journal.pone.0251974)
Supplement: S4 Table — Mod. Cla represents the percentage of individuals that performed each behavior. (DOCX) [file pone.0251974.s004.docx]

**Table S4.** Across-cluster distribution of behaviours, call types and sex. ‘Cla.Mod’ represents the percentage of individuals that performed each behaviour across clusters.

| **Variable** | | **Cluster 1** | **Cluster 2** | **Cluster 3** | **Cluster 4** | **Cluster 5** |
| --- | --- | --- | --- | --- | --- | --- |
|  |  | **Cla.Mod** | **Cla.Mod** | **Cla.Mod** | **Cla.Mod** | **Cla.Mod** |
| **Behaviour** | Defending from attack | 0 | 0 | 0 | 0 | **100** |
|  | Begging for food | **68.75** | 0 | 0 | 0 | 0 |
|  | Defending its food | 0 | 0 | **100** | 0 | 0 |
|  | Environment High | 0.91 | **99.09** | 0 | 0 | 0 |
|  | Environment low | **100** | 0 | 0 | 0 | 0 |
|  | Feeding | 0 | **100** | 0 | 0 | 0 |
|  | Interested at | **90.51** | 9.49 | 0 | 0 | 0 |
|  | Physical attack | 0 | 0 | 0 | 0 | **98.65** |
|  | Mating | 0 | 0 | 0 | 0 | **100** |
|  | Interacting or close by | 0 | 0 | 0 | **81.82** | 0 |
|  | Reject interaction or proximity | 0.76 | 5.34 | 9.16 | 0 | **84.73** |
|  | Social grooming | 0 | 0 | 0 | **98.04** | 0 |
|  | Social play | 0 | 0 | 0 | **39.13** | 0 |
|  | Soliciting Interaction | **99.09** | 0.90 | 0 | 0 | 0 |
| **Directed to** | People | **55.06** | **43.65** | 0.55 | 0.55 | 0.18 |
|  | Otter adjacent enclosure | 22.22 | 1.39 | 18.06 | 0 | **52.78** |
|  | Otter same enclosure | 21.34 | 9.94 | 5.26 | 17.25 | **46.2** |
|  | Itself | 0 | 0 | 0 | **100** | 0 |
| **Sex** | Female | 17.18 | **32.05** | 0 | 11.97 | **35.13** |
|  | Male | **67.34** | **24.61** | 0 | 1.34 | 3.36 |
| **Call type** | Chirp | **92.65** | 2.94 | 0 | 0 | 0 |
|  | Squeak | 10.45 | 0 | 0 | **89.55** | 0 |
|  | Chuckle | **78.96** | 19.80 | 0 | 1.24 | 0 |
|  | Growl | 0 | 0 | **100** | 0 | 0 |
|  | Hah | 0 | **98.47** | 1.01 | 0 | 0.50 |
|  | Scream | 0.49 | 0 | 0 | 0 | **97.51** |
