# Supplementary material for: Airborne vocal communication in adult neotropical otters (Lontra longicaudis)
Source: PLoS One. 2021 May 26;16(5):e0251974. doi: 10.1371/journal.pone.0251974 (PMC8153427; doi:10.1371/journal.pone.0251974)
Supplement: S5 Table — (DOCX) [file pone.0251974.s005.docx]

**Table S5.** Varimax normalized Principal Components Analysis showing principal components with eigen values greater than one and loadings matrix for 12 acoustic parameters in Tonal calls of otters.

|  | **Principal Components** | | |
| --- | --- | --- | --- |
| **Acoustic Parameter** | 1 | 2 | 3 |
| Mean Frequency (Hz) | **0.78** | 0.42 | 0.42 |
| Minimum Frequency (Hz) | **0.58** | 0.46 | 0.56 |
| Maximum Frequency (Hz) | **0.9** | 0.26 | 0.3 |
| Dominant Frequency (Hz) | 0.32 | **0.64** | -0.15 |
| Standard deviation F_0_ (Hz) | **0.92** | -0.04 | -0.01 |
| Initial F_0_ (Hz) | **0.88** | -0.02 | 0.35 |
| Mid-point F_0_ (Hz) | **0.83** | 0.24 | 0.42 |
| End F_0_ (Hz) | **0.7** | 0.57 | 0.36 |
| Slope First Half (Hz) | -0.24 | **0.86** | 0.09 |
| Slope Second Half (Hz) | **-0.4** | -0.09 | -0.34 |
| Duration (Sec) | -0.09 | 0.08 | **-0.9** |
| F_0_ Range (Hz) | **0.94** | -0.02 | -0.05 |
| **Proportion of explained variance** | 0.59 | 0.21 | 0.20 |
| **Cumulative proportion** | 0.59 | 0.80 | 1 |
