# Supplementary material for: Airborne vocal communication in adult neotropical otters (Lontra longicaudis)
Source: PLoS One. 2021 May 26;16(5):e0251974. doi: 10.1371/journal.pone.0251974 (PMC8153427; doi:10.1371/journal.pone.0251974)
Supplement: S6 Table — (DOCX) [file pone.0251974.s006.docx]

**Table S6.** Summarized results of Kruskall-Wallis test comparing acoustic parameters (PC1, PC2 and PC3) of tonal call types. P-values are also shown in Pairwise comparisons between call types using Wilcoxon rank sum tests with Bonferroni correction.

| **Comparisson** | **PC1** | | | **PC2** | | | **PC3** | | |
| --- | --- | --- | --- | --- | --- | --- | --- | --- | --- |
|  | **X^2^** | **df** | **p-value** | **X^2^** | **df** | **p-value** | **X^2^** | **df** | **p-value** |
| Chirp-Squeak | 68 | 3 | **<0.001** | 26 | 3 | 0.09 | 38 | 3 | **0.03** |
| Chirp-Chuckle |  |  | **<0.001** |  |  | **<0.001** |  |  | **<0.001** |
| Chirp-Growl |  |  | **<0.001** |  |  | 0.84 |  |  | **0.001** |
| Squeak-Chuckle |  |  | **0.03** |  |  | **0.03** |  |  | **<0.001** |
| Squeak-Growl |  |  | 0.41 |  |  | 0.19 |  |  | **0.01** |
| Chuckle-Growl |  |  | 0.71 |  |  | **0.001** |  |  | **<0.001** |
