# Supplementary material for: Airborne vocal communication in adult neotropical otters (Lontra longicaudis)
Source: PLoS One. 2021 May 26;16(5):e0251974. doi: 10.1371/journal.pone.0251974 (PMC8153427; doi:10.1371/journal.pone.0251974)
Supplement: S7 Table — (DOCX) [file pone.0251974.s007.docx]

**Table S7.** Varimax normalized Principal Components Analysis showing principal components with eigen values greater than one and loadings matrix for four acoustic parameters in NLP calls of otters.

|  | **Principal Components** | |  |
| --- | --- | --- | --- |
| **Acoustic Parameter** | 1 | 2 | |
| Dominant Frequency (Hz) | 0.2 | **0.554** | |
| Duration (Sec) | **0.842** | 0.47 | |
| Tonal duration (Sec) | -0.181 | **0.906** | |
| NLP duration (Sec) | **0.975** | -0.194 | |
| **Prop. of explained variance** | 0.56 | 0.44 | |
| **Cumulative proportion** | 0.56 | 1 | |
