# Supplementary material for: Airborne vocal communication in adult neotropical otters (Lontra longicaudis)
Source: PLoS One. 2021 May 26;16(5):e0251974. doi: 10.1371/journal.pone.0251974 (PMC8153427; doi:10.1371/journal.pone.0251974)
Supplement: S8 Table — (DOCX) [file pone.0251974.s008.docx]

**Table S8.** Summarized results of Kruskall-Wallis test comparing acoustic parameters (PC1 and PC2) of NLP call types.

| **Comparisson** | **PC1** | | | **PC2** | | |
| --- | --- | --- | --- | --- | --- | --- |
|  | **X^2^** | **df** | **p-value** | **X^2^** | **df** | **p-value** |
| Hah-Scream | 12 | 1 | **<0.001** | 42 | 1 | **<0.001** |
